# The age-related effects on orthodontic tooth movement and the surrounding periodontal environment

**DOI:** 10.3389/fphys.2024.1460168

**Published:** 2024-09-06

**Authors:** Jiayi Wang, Yiping Huang, Feng Chen, Weiran Li

**Affiliations:** ^1^ Department of Orthodontics, Peking University School and Hospital of Stomatology, National Center for Stomatology, National Clinical Research Center for Oral Diseases, National Engineering Research Center of Oral Biomaterials and Digital Medical Devices, Beijing Key Laboratory of Digital Stomatology, NHC Key Laboratory of Digital Stomatology, NMPA Key Laboratory for Dental Materials, Beijing, China; ^2^ National Center of Stomatology, National Clinical Research Center for Oral Diseases, National Engineering Laboratory for Digital and Material Technology of Stomatology, Beijing Key Laboratory for Digital Stomatology, Research Center of Engineering and Technology for Computerized Dentistry Ministry of Health, NMPA Key Laboratory for Dental Materials, Beijing, China; ^3^ Central laboratory, Peking University School and Hospital of Stomatology, Beijing, China

**Keywords:** orthodontic tooth movement, aging, age-related changes, periodontal ligament, alveolar bone, tooth movement speed

## Abstract

Orthodontic treatment in adults is often related to longer treatment time as well as higher periodontal risks compared to adolescents. The aim of this review is to explore the influence of age-related chages on orthodontic tooth movement (OTM) from macro and micro perspectives. Adults tend to show slower tooth movement speed compared to adolescence, especially during the early phase. Under orthodontic forces, the biological responses of the periodontal ligament (PDL) and alveolar bone is different between adult and adolescents. The adult PDL shows extended disorganization time, increased cell senescence, less cell signaling and a more inflammatory microenvironment than the adolescent PDL. In addition, the blood vessel surface area is reduced during the late movement phase, and fiber elasticity decreases. At the same time, adult alveolar bone shows a higher density, as well as a reduced osteoblast and osteoclast activation, under orthodontic forces. The local cytokine expression also differs between adults and adolescents. Side-effects, such as excessive root resorption, greater orthodontic pain, and reduced pulpal blood flow, also occur more frequently in adults than in adolescents.

## 1 Introduction

Malocclusion is the deviation from normal or ideal occlusion, which can affect oral health, function, and facial aesthetics of patients. Malocclusion occurs in all populations; its prevalence among children and adolescents ranges from 48% to 83.5% (Lombardo et al., 2020; Yin et al., 2023), and 45.6%–68% in the adult population (Jonsson et al., 2007; Claudino and Traebert, 2013; Asiri et al., 2019). With the recent focus on quality of life and the increased emphasis on appearance, adults pay more attention to having an “ideal” appearance. Therefore, the demand for orthodontic treatment in adults has been substantially increasing.

**FIGURE 1:**
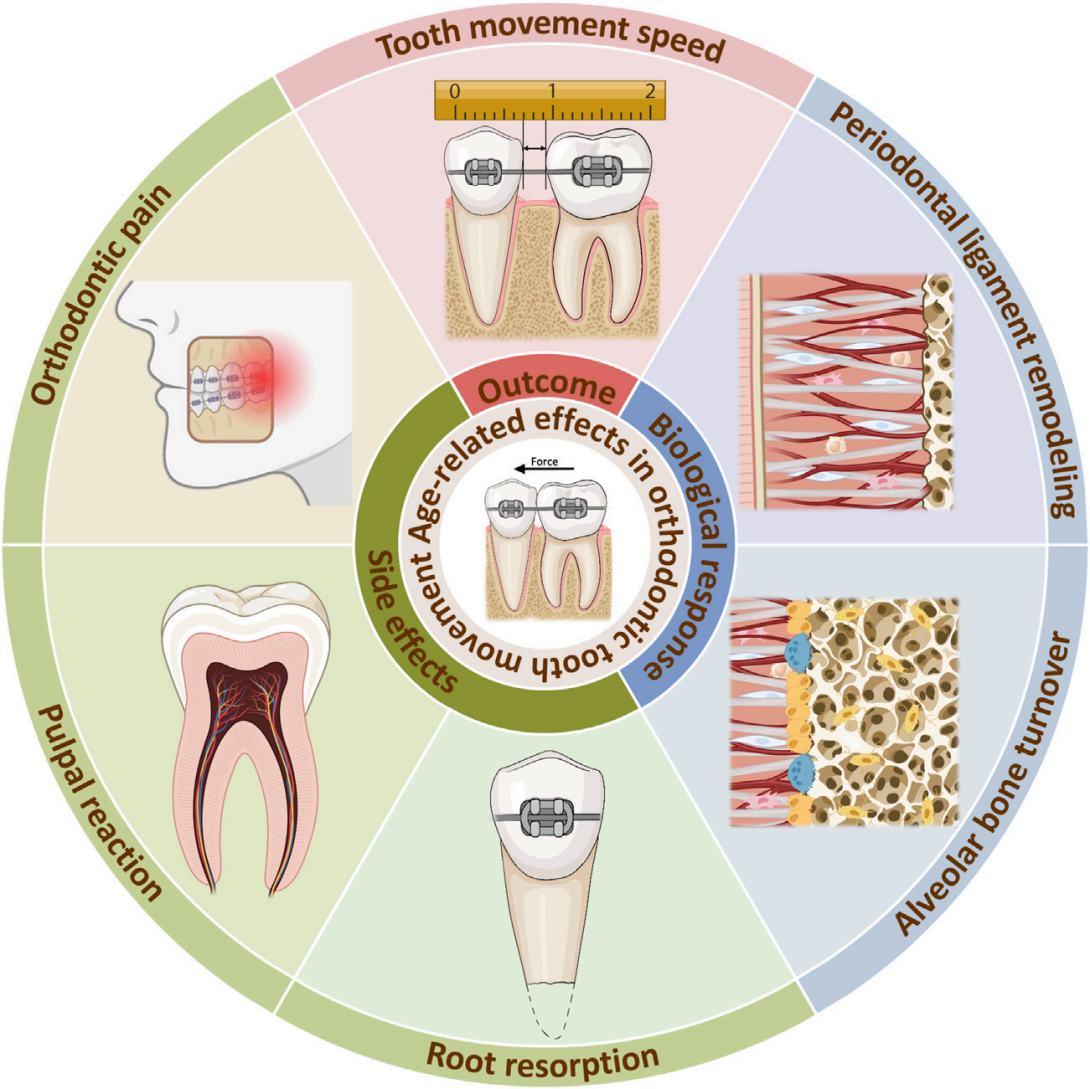
Age-related effects in orthodontic tooth movement. Age-related changes occurs during orthodontic treatment, reducing tooth movement speed. The internal reason can be explained by alterations of biological responses in the PDL and alveolar bone. Meanwhile, side effects such as orthodontic pain, pulpal reaction, root resorption can be aggrevated. Created with BioRender.com.

Clinically, orthodontic treatment in adults is often related to longer treatment times, as well as higher periodontal risks than in adolescents, which may hinder the application of orthodontic treatment in adults (Ren et al., 2003; Alikhani et al., 2018). This is related to the maturation and aging process. As the human body experience aging, there is a time-dependent decline in function, which is the result of the cumulative effect of various molecular and cellular damages at the microscopic level (Li et al., 2016; Calcinotto et al., 2019; Mohanakumar et al., 2021). Thus, tissue properties and biological responses are altered. As a biological process, orthodontic tooth movement (OTM) is also impacted by age-related changes. During orthodontic treatment, the tooth moves within the alveolar bone under mechanical forces. This process is essentially a localized aseptic inflammatory response in the periodontal ligament (PDL) and alveolar bone, mediated by mechanical forces, which results in bone resorption on the pressure side and bone deposition on the tension side (Li et al., 2021). The senescence related changes of the tooth, surrounding periodontium and the alveolar bone may all contribute to the age-related changes of OTM (Figure 1).

This review focuses on the difference in biological reactions to orthodontic treatment between adults and adolescents. With a comprehensive understanding of the age-related biologic and mechanistic changes in the OTM process, key regulatory targets may be identified. This can improve the clinical outcomes of orthodontic treatment in adults and provide insight on future research directions. Hence, the aim of this review is to explore the influence of age-related changes on OTM from macro and micro perspectives, as well as the involved inner mechanisms.

## 2 Reduced tooth movement speed in adults during orthodontic treatment

The movement speed is an important factor in OTM as it determines the course of the treatment. Many researchers have discovered that tooth movement speed in adult human is slower than that in adolescents. For instance, during the maxillary canine distalization, after 7 days of traction, a longer movement distance of canines was found for adolescents than for adults (Kawasaki et al., 2006). The faster movement of canines in adolescents continues even up to 56 days of canine distalization, especially during the 28–56 days period (Alikhani et al., 2018). The same result was found in animal models. Molar movement distance in old rats was significantly smaller than that observed in young rats over a total of 12 weeks. This tooth movement speed appears to be significantly higher in young rats than in older rats during the early phase (0–3 weeks), but gradually becomes similar during the late phase (4–12 weeks) (Ren et al., 2003). Similar results of tooth movement being more rapid in adolescents than in adults were confirmed by many in both human and animals (Kyomen and Tanne, 1997; Von Böhl et al., 2016). However, Li’s study found a tendency for decreasing molar movement distance in old (18–20 weeks) rats compared to young (4–5 weeks) rats, after applying 7 days of orthodontic force; however, no significant difference was found (Li et al., 2016). This may be due to the minimal age gap between the two groups or the short duration of the experiment.

When teeth are subjected to orthodontic forces, the physiological process of their movement can be divided into four phases according to speed: initial phase, lag phase, acceleration phase, and linear phase (Pilon et al., 1996). In the initial phase, the tooth undergoes an instantaneous movement in its socket, pressuring the PDL. Therefore, its movement distance is limited by the compression and viscoelastic properties of the PDL. Subsequently, OTM enters the lag phase, in which its speed decelerates and tooth movement arrests. This could be attributed to the hyalinization of the PDL and blockage of the alveolar bone. Under compressive force, phagocytes and osteoclasts locally accumulated, removing hyalinized areas and initiating PDL turnover and bone remodeling. As accumulation and activity of these cells increase, the tooth movement speed elevates, leading to the acceleration phase. When local cellular activities reach homeostasis, PDL turnover and bone remodeling achieve maximum capacity, resulting in constant tooth movement speed as in the linear phase.

As described above, a significant faster tooth movement velocity occurred in young rats only during the early phase of OTM (Ren et al., 2003), suggesting that the biological difference between adolescences and adults takes place at the early phase. This may be related to the speed of the initial response of the PDL and alveolar bone cells.

## 3 Reduced regenerative capacity and enhanced inflammatory response in aged PDL during orthodontic treatment

**FIGURE 2:**
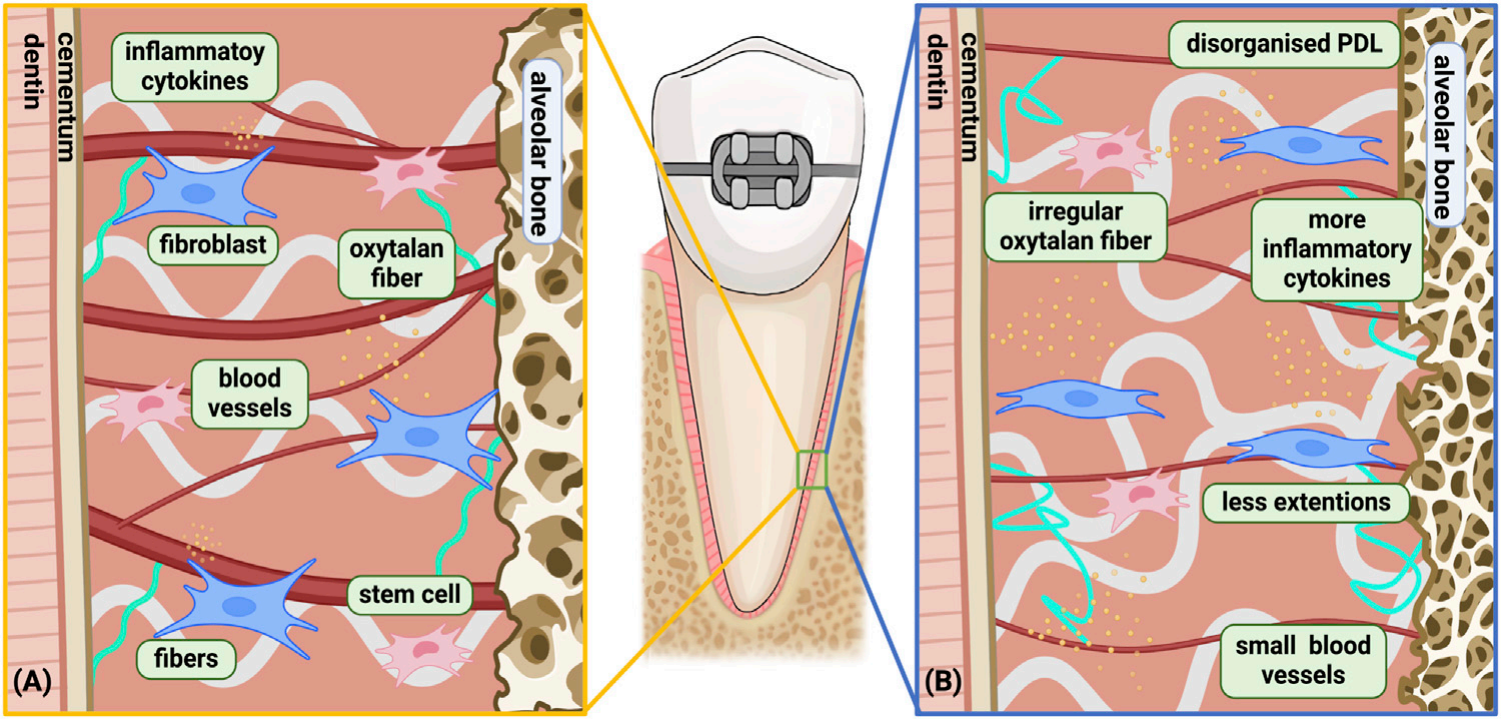
Age-related changes in the biological response of PDL under orthodontic force. **(A)** Young PDLC under orthodontic force; **(B)** aged PDLC under orthodontic force. Differences between the Young and Aged PDLC was shown: a) Slower biological reaction and recovery with increased disorganization time; b) Cell senescence; c) More inflammatory microenvironment; d) Fewer extensions in cells leading to deteriorated signaling activity; e) More complex and irregular oxytalan fibers with loss of elasticity; f) Reduced blood vessel surface area during late phase, with solely proliferation of small blood vessels instead of large blood vessel. Among the above differences, *a* only occurs on the compression side, while *b, c, d, e, f* occurs on both the compression side and the tension side. Created with BioRender.com.

As the connective tissue between teeth and alveolar bone, the PDL is composed of a heterogeneous cell population, including fibroblastic lineages, osteoblastic lineages, multipotent stem cells etc. as well as fibrous structures and blood vessels (Nanci and Bosshardt, 2006). The PDL plays a crucial role in OTM with the ability of mechanotransduction, cellular signaling, self-renewal and direct or indirect regulation of periodontal tissue remodeling. Various components and their function experience noticeable changes with aging (Figure 2) (Li et al., 2021). Cell proliferation ability, migratory potential and differentiation capacity declines as age development occurs (Lim et al., 2014; Chen et al., 2012). Meanwhile, cytokine expression is also altered in adults (Table 1) (Wu et al., 2015). Other components, including blood vessels and collagen content, undergo degenerative changes influenced by aging (Sims et al., 1996; Lim et al., 2014).

**TABLE 1:** Molecule changes in OTM between adults and adolescents.

Molecule	Type	Model	Subject/participant age	Observation time	Sorce	Age-related differential expression in OTM	Possible function	Reference
SA-β-GAL	Protein	Human OTM	Young: 12–20 years Adult: 35–50 years	28 days	PDLCs	Higher in adults before and after OTM. Both groups showed reduction during OTM, being more significant in adults	Lysosomal enzyme; marker for cell scenesence	Mohanakumar et al. (2021)
α-Klotho	Protein	Human OTM	Young: 12–20 years Adult: 35–50 years	28 days	PDLCs	Lower in adults before and after OTM. Both increased during OTM, with a greater change in young group	Anti-scenesence	Mohanakumar et al. (2021)
PEG2	mRNA	Human OTM	Young: 16.4 ± 2.78 years Adult: 40.9 ± 3.99 years	7, 14, 28 days	PDLCs	Higher in adults at baseline and day 7, 14, 28. Both group experienced a significant increase	Inflammatory cytokine Osteoclastogenic mediator Pain mediator	George et al. (2020)
	Protein	Human OTM	Young: 13 ± 2.1 years Adult: 24 ± 2.1 years	2, 21, 28 days	GCF	Higher in adults at base line and day 28. Young group experienced peak at day 21		Chibebe et al. (2010)
			Young: 11 ± 0.7 years Adult: 24 ± 1.6 years	24 h	GCF	Higher in adults at base line and 24 h. Both group experienced a significant increase		Ren et al. (2002)
		Cell compression	9–50 years	48 h	PDLCs	PGE2 product in response to compressive force, increase of production is age-dependent		Mayahara et al. (2007)
COX-2	mRNA	Cell compression	9–50 years	3,6,12,24.48 h	PDLCs	Cox-2 level upregulates with time, increase of production is age-dependent. Increase is especially significant after age 36	Induced from mechanical stress Participates in PGE2 synthesis	Mayahara et al. (2007)
IL-1β	mRNA	Human OTM	Young: 16.4 ± 2.78 years Adult: 40.9 ± 3.99 years	7, 14, 28 days	PDLCs	Higher in adults at baseline and day 7, 14, 28. Both group experienced a significant increase	Inflammatory cytokine Osteoclastogenic promoter	George et al. (2020)
	Protein	Human OTM	Young: 13.3 ± 0.9 years Adult: 31 ± 5.5 years	1, 7, 14, 28 days	GCF	Higher in adults at day 7, 14, both group peaked at day 1		Alikhani et al. (2018)
IL-6	Protein	Human OTM	Young: 11 ± 0.7 years Adult: 24 ± 1.6 years	24 h	GCF	Higher in adults at base line and 24 h. Only young group experienced a significant increase	Inflammatory cytokine Tissue remodeling Pain mediator	Ren et al. (2002)
GM-CSF	Protein	Human OTM	Young: 11 ± 0.7 years Adult: 24 ± 1.6 years	24 h	GCF	Higher in adults at base line and 24 h. Only young group experienced a significant increase	Inflammatory cytokine	Ren et al. (2002)
RANKL	mRNA	Human OTM	Young: 16.4 ± 2.78 years Adult: 40.9 ± 3.99 years	7, 14, 28 days	PDLCs	Lower in adults at day 28. Both group experienced a non-significant increase	Improtant component of the NF-κB pathway Activates osteoclastic activities	George et al. (2020)
	Protein	Rat OTM	Young: 4–5 weeks Adult: 18–20 weeks	7 days	parrafin slides	No significant difference between groups at baseline and day 7 both on pressure and tension side. Both group experienced a significant increase		Li et al. (2016)
		Human OTM	Young: 15.1 ± 2.8 years Adult: 31 ± 5.5 years	1, 24, 168 h	GCF	Lower in adults at 24 h. RANKL expression only showed significant increase at 24 h		Kawasaki et al. (2006)
			Young: 13.3 ± 0.9 years Adult: 31 ± 3.6 years	1, 7, 14, 28 days	GCF	Higher in adults at day 1, 7, 14, both group peaked at day 7		Alikhani et al. (2018)
OPG	mRNA	Human OTM	Young: 16.4 ± 2.78 years Adult: 40.9 ± 3.99 years	7, 14, 28 days	PDLCs	Lower in adults at day 7, and higher at day 28. Only adults showed significant increase in OPG expression from day 7 to day 28		George et al. (2020)
	Protein	Human OTM	Young: 15.1 ± 2.8 years Adult: 31 ± 5.5 years	1, 24, 168 h	GCF	Lower in adults at baseline, 1, 168 h, higher in adults at 24 h. Both group experienced significant reduction at 24 h	RANKL antagonist; Osteoclastogenesis inhibitor	Kawasaki et al. (2006)
TNF-α	Protein	Human OTM	Young: 13.3 ± 0.9 years Adult: 31 ± 5.5 years	1, 7, 14, 28 days	GCF	Higher in adults at day 1, 7. Both group peaked at day 1	Inflammatory cytokine Osteoclastogenic promotor	Alikhani et al. (2018)
CCL2	Protein	Human OTM	Young: 13.3 ± 0.9 years Adult: 31 ± 5.5 years	1, 7, 14, 28 days	GCF	Higher in adults at day 1, 7. Both group peaked at day 1	Chemokine Recruit and activate osteoclasts	Alikhani et al. (2018)
MMP9	Protein	Human OTM	Young: 13.3 ± 0.9 years Adult: 31 ± 5.5 years	1, 7, 14, 28 days	GCF	Higher in adults at day 1, 7, 14. Both group peaked at day 1 and reduced to baseline level at day 28	Inflammatory cytokine Mediate proteolysis in bone resorption Tissue remodeling	Alikhani et al. (2018)
ALP	mRNA	Human OTM	Young: 16.4 ± 2.78 years Adult: 40.9 ± 3.99 years	7, 14, 28 days	PDLCs	Lower in adults at day 28. Both group showed a trend of increased ALP expression on day 7, 14, 28 while only significant on day 28	Bone formation	George et al. (2020)
BGLAP	mRNA	Human OTM	Young: 16.4 ± 2.78 years Adult: 40.9 ± 3.99 years	7, 14, 28 days	PDLCs	Lower in adults at day 14. Both group experienced a non-significant increase	Osteoblast marker Bone formation	George et al. (2020)

### 3.1 Age-related changes in periodontal ligament cells (PDLCs)

Age-related changes in the PDL have also been observed under orthodontic forces. When tooth movement occurs, surrounding PDLCs are compressed on the pressure side, forming degenerated tissue while they extended on the tension side (Shimpo et al., 2003). From a phenotypic perspective, mouse PDL showed little difference between aged and young rats on the tension side, with abundant collagen fibers, PDLCs and osteoblasts (Ren et al., 2008c). However, a slower biological reaction and recovery were found in the PDL of aged rats on the pressure side. While young rats experienced PDL disorganization after 1–2 weeks of force application and rearranged at 8–12 weeks, the disorganized status of the PDL extended to 12 weeks in aged rats, as recovery was not observed within the 12-week trial (Ren et al., 2008c). This corresponds with higher proliferative activity of PDLCs in young rats than in adult rats during the first week of OTM on both the pressure side and tension side (Kyomen and Tanne, 1997). The reduction in physiological response and reconstruction capability of the PDL with age corresponds with the decreased movement speed.

Furthermore, adult human PDLCs (hPDLCs) exhibited increased senescence with higher beta-galactosidase activity as well as lower α-Klotho expression (Mohanakumar et al., 2021). Senescence-associated beta-galactosidase (SA-β-GAL) serves as a commonly used biomarker to detect and quantify the aging status of cells, with its activity significantly increases in senescent cells. α-Klotho is the co-receptor for Fibroblast Growth Factor-23 (FGF23) and is an important anti-aging protein associated with phosphate regulation, vitamin D metabolism, oxidative stress reduction and insulin signaling. During orthodontic movement, both adult and young hPDLCs showed decreased SA-β-GAL level and increased α-Klotho expression. However, adult hPDLCs still had higher SA-β-GAL level and lower α-Klotho expression compared with young hPDLCs(Mohanakumar et al., 2021). Furthermore, the SA-β-GAL level in adult PDLCs ascended before descending, with the peak at day 7, while it exhibited a straightforward decrease in young hPDLCs (George et al., 2020). Therefore, adult hPDLCs still exhibit higher levels of senescence and lower anti-aging protein expression compared to younger cells, even during orthodontic movement.

Inflammatory changes also take place in adult hPDLCs, with a significant increase in PGE2 and IL-1β mRNA expression level higher than young hPDLCs during the first month of OTM (Mayahara et al., 2007; George et al., 2020). PGE2 is a critical inflammatory cytokine and osteoclastogenic mediator during the OTM process, which is expressed in hPDLCs under mechanotransduction and related with osteoclastogenesis and regulation of osteo-related cytokines (Kang et al., 2010; Ullrich et al., 2019). IL-1β is also associated with acute inflammation and bone resorption during tooth movement (Alhashimi et al., 2001; Kapoor et al., 2014). These differences suggest a stronger inflammatory microenvironment under mechanical stress in adult PDL than in young PDL, along with more catabolic changes. Subsequently, relating changes occurred in PGE2 and IL-1β concentration in the gingival cervical fluid (GCF) of adult and young patients. PGE2 concentration experiences a significant increase followed by a decline with the peak at 21 days of OTM in young patients, while it maintains a high concentration in adult patients (Ren et al., 2002; Chibebe et al., 2010). Additionally, GCF PGE2 concentration of young patients is lower than adult patients at all times except at peak, demonstrating a more inflammatory periodontal microenvironment in adults at all time. Likewise, IL-1β concentration is significantly higher in adult human GCF after 7 days of OTM(Alikhani et al., 2018). PGE2 can be synthesized by COX-2, a prostaglandin endoperoxidase, which is stimulated under mechanical stress and a critical component of the orthodontic movement process (Mitsui et al., 2005; de Carlos et al., 2006). A rapid increase in COX-2 mRNA expression and cell expression ratio after 48 h of compressive force has also been found in adult hPDLCs compared with young hPDLCs(Mayahara et al., 2007). IL-6 and GM-CSF are also important inflammatory mediators involved in the orthodontic process (Bakker et al., 2014; Nunes et al., 2017; Feng et al., 2022). Similarly, IL-6 and GM-CSF concentrations were higher in adult human GCF than adolescents during OTM (Ren et al., 2002). Adult hPDLCs experience more pronounced inflammatory responses and catabolic changes, suggesting a more inflammatory environment in adult PDL under mechanical stress.

### 3.2 Age-related changes in periodontal fibers and blood vessels

Age-related changes also take place in the extracellular matrix, including fibers and the vascularis. Oxytalan fibers is an important component of the PDL, as a kind of elastic fiber, it is composed of microfibrils and can provide tissue resilience and flexibility. Oxytalan fibers are suggested to support and regulate the vascular system, guide fibroblast migration and contribute to the mechanical properties of the PDL (Strydom et al., 2012). Under tension strain, microfibril synthesis is upregulated in PDL fibroblasts, resulting in oxytalan fiber bundle thickening (Tsuruga et al., 2009). It is believed that oxytalan fibers is not altered by age changes in the PDL under standard physiological conditions (Moxham and Evans, 1995). However, previous studies found oxytalan fibers in aged rats exhibiting different reactions under orthodontic force from those in young rats. More oxytalan fibers prevailed around blood vessels, oxytalan meshwork were more complex and irregular, indicating a loss of elasticity under orthodontic force in rats (Chantawiboonchai et al., 1999).

The characteristics of blood vessels in the PDL also undergo age-related changes. As important constituents of the PDL, blood vessels provide oxygen, nutrients, immune cells for the tissue reconstitution during the orthodontic process (Li et al., 2021). Under mechanical pressure, blood vessels are compressed and deformed, creating a hypoxic environment along with circulating immune cell migration, starting a sterile inflammatory response while starting the osteoclastic process. Vascular endothelial cells also stimulate osteoblast differentiation through Runx2 expression (Kohno et al., 2003). Ren et al. (2008b) measured the mean surface area of the periodontal blood vessels in mice. Adult mice presented higher blood vessel surface area than young mice without stress and during the initial phase (1–2 weeks) of orthodontic treatment. However, when moving on to the late phase (2–8 weeks), the blood vessel surface area exhibited a significant decrease, reaching a level similar to that of young mice. Meantime, the numbers of PDL blood vessels also increased during the late phase of OTM in all mice. Large (surface area > 500 µm^2^) and small (surface area < 500 µm^2^) blood vessels increased in young mice, while only small blood vessels increased in old mice. This discrepancy has not been clarified and still need more comprehensive research.

## 4 Delayed bone remodeling response in adults during orthodontic treatment

The ability of bone remodeling is crucial in the orthodontic procedure as movement of tooth depends on the resorption of alveolar bone on the pressure side and deposition on the tension side. In order to initiate bone remodeling, osteoblast and osteoclast activation and differentiation is fundamental. This relies on a cell-to-cell communications network which consists of PDLCs, osteocytes, osteoblasts, osteoclasts, immune cells as well as various cytokines (Li et al., 2021). Foremost, after orthodontic force application, with signals transduced through the membrane receptors and ion channels, a swift response of mechanosensitive PDLCs come into action (Jin et al., 2015; Chang et al., 2020; Jin et al., 2020). Subsequently, secreting cytokines are released into the extra cellular matrix. The expression of TNF-related ligand RANKL as well as the ratio with its antagonist OPG are increased, indicating the beginning of catabolic procedures in the alveolar bone (Kawasaki et al., 2006; Shen et al., 2020). Meanwhile, the activation of osteoclasts is also mediated by other inflammatory cytokines. The expression of the interleukin family (IL-1β, Il-2, Il-6, Il-8 etc.), TNF-α, IFN-γ were all upregulated as a result of force application, therefore regulating the signaling of several osteoclastogenic pathways (Grant et al., 2013; Kapoor et al., 2014). Meanwhile, activation of osteoblasts are also found with the elevation of its specific marker Osteocalcin, symbolizing the anabolic process of the alveolar bone (Teixeira et al., 2017).

### 4.1 Slower osteoclast activation and regulated cytokine expression in aged orthodontic process

**FIGURE 3:**
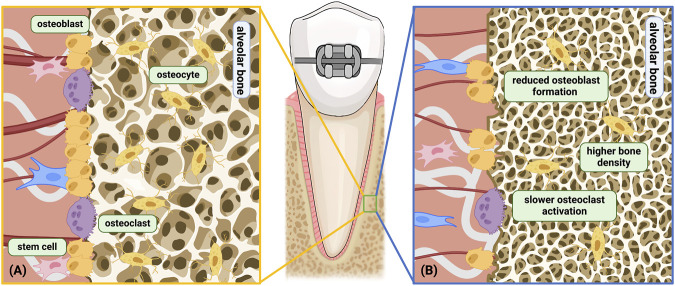
Age-related changes related to the alveolar bone under orthodontic force. **(A)** Young alveolar bone remodeling under orthodontic force; **(B)** aged alveolar bone remodeling under orthodontic force. Differences shown above: a) Higher bone density; b) Slower osteoclast activation and decreased cell sensitivity; c) Reduced osteoblast differentiation and formation; d) Hindered bone turnover. Among the above differences, *b* only occurs on the compression side, while *a, c, d* occurs on both the compression side and the tension side. Created with BioRender.com.

Compared with adolescents, the alveolar bone density in adults is often higher (Bridges et al., 1988), especially during mid-age, both in human and animals (Boskey and Coleman, 2010). However, the number of osteoclasts in adult rats is lower than young rats during the initial phase of tooth movement (0–2 weeks), which corresponds to the faster movement speed in young rats during early phase (Ren et al., 2003). During the linear phase, the number decreases in young rats and increases in adult rats, gradually resulting in higher osteoclast numbers in adult rats (Ren et al., 2005; Li et al., 2016). Compared to adults, adolescents have a more rapid activation and recruitment of osteoclasts and may initiate the bone resorption process earlier (Figure 3). Similarly, after removal of orthodontic forces, the regression of osteoclast number is much faster in young rats than adult rats (Li et al., 2016).

The activation and recruitment of osteoclasts relies on cytokines, especially RANKL and M-CSF, which activates the NF-κB pathway and the MAPK phosphorylation cascade (Brooks et al., 2011; Nakashima et al., 2011; Kitaura et al., 2020). RANKL can be expressed by osteoblasts, osteocytes, PDLCs, and some immune cells including T cells, B cells. When RANKL is ablated from PDLCs and bone lining cells, the formation of osteoclasts was blocked and tooth movement speed decreased (Yang et al., 2018). Currently, the changes in RANKL expression with age are still unclear and vary with patients. During the initial and linage phases, the RANKL expression in PDLCs increased in both young and adult patients. However, no significant differences was found between the two, with the expression being slightly higher in young patients (George et al., 2020). Similar results were found in animals, with a non-significant RANKL expression between young and adult rats on both the tension and the pressure side during OTM (Li et al., 2016). The changes in RANKL levels in gingival crevicular fluid are more unstable. Kawasaki et al. (2006) have found increased RANKL level after tooth movement in adolescent gingival crevicular fluid significantly higher than adults in human, while Alikhani et al. (2018) believe that the increase in RANKL levels in adult human is higher. The inconsistencies in experimental results may derive from research heterogenicity and difference in methodology. In addition, cytokine levels in gingival crevicular fluid can be influenced by multiple factors, including probing depth, oral hygiene, degree of gingival inflammation, periodontal treatment history, sampling area etc. (Hatipoğlu et al., 2007; Wassall and Preshaw, 2016; López Roldán et al., 2020).

OPG inhibits the activation of osteoclasts and the process of bone resorption by competitively binding to RANKL, preventing the interaction between RANKL and its receptor RANK (Kanzaki et al., 2001; Eriksen, 2010). During OTM, OPG expression level maintained a low expression pattern in adolescent hPDLCs, while its levels elevated in adult hPDLCs (George et al., 2020). Meantime, OPG level in the human GCF was downregulated during OTM, with a smaller reduction in adult GCF than adolescents (Kawasaki et al., 2006). Although the regulation tendency is different between the two studies, the OPG level is always higher in adults, promoting bone formation while limiting the osteoclastogenic rate of RANKL. The RANKL⁄OPG ratio is also an indicator of bone remodeling capability (Yamaguchi, 2009). The adult RANKL⁄OPG ratio in hPDLC expression as well as in the human GCF was relatively inferior to that in adolescents during OTM, especially during the initial phase (Kawasaki et al., 2006; George et al., 2020).

The concentration of TNF-α and CCL2 peaked in the GCF after 1 day of OTM in human, and soon returned to baseline at day 7 in adolescents, while maintaining a high level in adults till day 14 (Alikhani et al., 2018). Similarly, MMP9 concentration in the human GCF peaked at day 1 and returned to baseline at day 28 in both adults and adolescents, with a significantly higher concentration in adults during OTM, though no difference were found at baseline (Alikhani et al., 2018). TNF-α is a proinflammatory cytokines and can promote osteoclast formation through activating the NF-κB pathway (Marahleh et al., 2019; Noguchi et al., 2020). CCL2 belongs to the chemokine family, and promotes chemotaxis, recruitment and activation of osteoclasts (Taddei et al., 2012). Meanwhile, MMP9 is a kind of matrix metalloproteinase secreted by osteoclasts, and participates in mediation of proteolysis (Henriksen et al., 2011). It is also a critical participant in the OTM process, depletion of MMP9 inhibits tooth movement (Holliday et al., 2003). Conversely, adult rats have less osteoclasts than young rats during the initial phase of OTM(Ren et al., 2005; Li et al., 2016).

### 4.2 Reduced osteoblast differentiation and formation in aged orthodontic process

Besides bone resorption, bone deposition as an anabolic process is also an important part of bone remodeling. ALP and Osteocalcin are classic markers for osteogenic activity (Wei and Karsenty, 2015; Makris et al., 2023). ALP expression in human was significantly upregulated in adolescents during OTM, while remaining essentially stable in adults, leading to a significant difference between the two on day 28 (George et al., 2020). Osteocalcin, encoded by the BGLAP gene and solely produced by osteoblasts, plays a significant role in osteogenesis and bone mineralization. BGLAP expression experienced a delayed increase in adults during OTM. Its expression was upregulated in adolescent hPDLCs throughout the initial and late phase, while only increased from day 14 to day 28 in adult hPDLCs (George et al., 2020).

## 5 Root resorption, pulpal reaction, local pain sensation and gingival reactions experience age-related changes in orthodontic movement

Orthodontic treatment is often accompanied by various side effects, including root resorption, pulp reactions, and local pain. These can affect the patient’s treatment experience and the teeth integrity. Moreover, the occurrence and severity of these side effects can be influenced by maturation and aging.

### 5.1 Increased root resorption in adults during tooth movement

Root resorption is a common and inevitable complication in orthodontic treatment. Mechanical loading on the periodontal tissue leads to degradation and hyalinization of the PDLCs and blood vessels, creating a sterile inflammatory environment (Lin et al., 2023), which subsequently activates odontoclasts and resorption of the superficial root cementum or even the underlying dentin (Jepsen et al., 2023). Ren et al. found a significant increase in average root resorption severity and max root resorption severity on the pressure side between the early phase (0–2 weeks) and late phase (4–12 weeks) of OTM in adult rats, while no difference was seen in young rats (Ren et al., 2008a). The root resorption severity was positively correlated with tooth movement duration and amount, while negatively correlated with tooth movement speed in rats (Ren et al., 2007). However, no difference was spotted respecting the incident of root resorption, average root resorption severity and max root resorption severity between adult and young rats during the 12 weeks of tooth movement (Ren et al., 2008a). Adults may experience more severe root resorption than adolescents as treatment time extends. The elevated expression of inflammatory cytokines plays a crucial role in root resorption, activating odontoclasts (Yamaguchi and Fukasawa, 2021; Lin et al., 2023).

### 5.2 Reduced pulpal flow in adults during tooth movement

Age-related changes also occur in the dental pulp. Due to the production of secondary dentin with aging, the pulp chamber narrows, followed by increased calcification within the dentinal tubules. Meanwhile, pulpal fibrosis, decreased cellular components, nerves and blood vessels, as well as declined proliferation and differentiation of mesenchymal stem cells occurs with pulpal aging (Maeda, 2020; de Farias and Rezende, 2023). The orthodontic process further causes age-related changes in the dental pulp. Adult rats experience a significantly aggravated ablation of dental cusps during OTM than young rats (Von Böhl et al., 2016). A thicker layer of predentin at the pulpal horn and pulp chamber roof is exhibited, though no significant difference of the thickness was observed compared to the control group. Reaction of pulpal blood flow to orthodontic treatment was different in adults and adolescents as well. Throughout the orthodontic process, the pulp blood flow in adults was consistently lower than in adolescents in human (Ersahan and Sabuncuoglu, 2018). The pulpal blood flow in both groups decreased within the first week of tooth movement. However, the pulpal blood flow returned to baseline by week three in adolescents, whereas in adults, it had not recovered at 1 month. The delayed pulpal blood flow recovery in adults may be related to hindered angiogenesis response. Angiogenesis response takes place in the dental pulp after initiation of OTM, especially in the crown third and root third of the pulp (Derringer et al., 1996). Amid this process, the release of VEGF, FGF-2, PDGF, TGF-β and EGF in response to orthodontic forces promotes the regeneration of micro-vessels, therefore leading to reconstruction of the local vascular network and the rebound of pulpal blood flow (Derringer and Linden, 2004; von Böhl et al., 2012).

### 5.3 Enhanced pain response in adults during tooth movement

Pain is often seen after orthodontic force application. Adult patients experience more pain and discomfort during tooth movement (Alikhani et al., 2018). This is related to the enhanced periodontal inflammatory responses in adults after force application, including vascular, cellular and chemical events (Long et al., 2016). Under orthodontic forces, the blood vessels are compressed and undergo hypoxia, with subsequently increased permeability, immune cells are locally recruited and infiltrates the PDL. Meanwhile, these PDLCs produce abundant inflammatory cytokines such as IL-1β, IL-6, CCL2, TNF-α, PGE2, generating orthodontic pain, which are more expressed in adult PDLCs (Yang et al., 2014; Gameiro et al., 2015; Long et al., 2016; Toyama et al., 2023).

### 5.4 Compromised gingival reactions among adult orthodontic patients

The gingiva is believed to experience remodeling during orthodontic treatment. Changes on the keratinized gingiva height, sulcus depth, papilla height and gingival invaginations may appear at the space closure sites in premolar extraction patients, there are also risks of gingival recession in human (Ramos de Faria et al., 2023; Alsulaimani and Qali, 2024; Arsić et al., 2024; Han et al., 2024; Seidel et al. 2022). As age increase, the soft tissue reaction towards orthodontic treatment varies. A more severe gingival recession is found in adult orthodontic patients, compared with adolescents (Alhaija et al., 2018). The causative agent may be the difference in periodontal hygiene, as dental calculus and stains are more prevalent in adult orthodontic patients, however, there is significantly more dental biofilms in children than adults (Mei et al., 2017; Alhaija et al., 2018). On the other hand, other periodontal indices showed no significant difference between adults and adolescents in human, including gingival index, plaque index, probing depth (Alhaija et al., 2018). Furthermore, gingival invaginations are more severe in adult patents with a larger invaginate volume, though the prevalence were identical between adults and adolescents (Stappert et al., 2018). Gingival invaginations are believed to promote relapse and impair periodontal health, thus this difference must be recognized (Gölz et al., 2011).

## 6 Interventions for acceleration of adult orthodontic treatment

The primary factor deterring adults from seeking orthodontic treatment is the extended treatment duration. Thus, interventions for acceleration of tooth movement are of high demand. Interventions for tooth movement acceleration can be classified into three types, including surgical approaches, physical stimuli and biological approaches.

The regional acceleratory phenomenon (RAP) is believed to be the mechanism of surgical mediated acceleration of OTM, which is the enhancement of local bone turnover (Frost, 1983; Wilcko et al., 2009; Hu and Li, 2022). When the cortical bone is surgically injured, osteoblasts and osteoclasts gather, boosting local bone turnover (Zhou et al., 2019; Erdenebat et al., 2022). Immune cells such as macrophages also participates during this process, while there are increased expression inflammatory cytokines such as TNF-α (Kinjo et al., 2022; Wang et al., 2022). The effect of RAP often last for 4 months and peaks at 1–2 months after surgery (Yaffe et al., 1994). Common surgical approaches include corticotomy, piezocision, micro-osteoperforations and periodontally accelerated osteogenic orthodontics (PAOO), which are all effective in inducing tooth movement acceleration (Keser and Naini, 2022). Compared with corticotomy, piezocision and micro-osteoperforations is less traumatic, while PAOO may maintain or improve the periodontal environment after treatment, and increase the range of tooth movement (Gao et al., 2021).

Though surgical approaches provide committed acceleratory effects, trauma cannot be avoided. Therefore, alternative physical or biological methods are still being sought. Physical stimuli are believed to achieve accelerated tooth movement. Light vibrational forces (LVF) and photobiomodulation (PBM) therapy are common techniques aiming to increase tooth movement speed. LVF is believed to promote osteogenesis, while stimulate expression of inflammatory cytokines and number of osteoclasts, thus expected to improve OTM speed (Nishimura et al., 2008; Slatkovska et al., 2011; Phusuntornsakul et al., 2018). However, the acceleratory effect of LVF is still controversial, meta-analysis uncovered that neither did LVF reduce total orthodontic treatment duration, nor did it increase tooth movement speed at the alignment stage or the space closure stage (El-Angbawi et al., 2023). Yet, other studies detect a raise in tooth movement speed (Leethanakul et al., 2016; Tangtanawat et al., 2023). PBM therapy, also known as low- level laser therapy (LLLT), is believed to activate osteoclasts, elevate inflammatory cytokine level and modulate bone metabolism through local laser irradiation (Yamaguchi et al., 2010; Altan et al., 2012; Hsu et al., 2018). There is sufficient proof that PBM can stimulate tooth movement and reduce treatment duration, especially during the initial alignment stage (Cruz et al., 2004; Kau et al., 2013; El-Angbawi et al., 2023).

Other biological approaches have been tested for promoting tooth movement, one of the clinically applied methods is the use platelet-rich fibrin (PRF). It is believed that PRF may increase the concentration of bone metabolic related proteins including RANKL, MMP-8, TNF-α, and Il-1β, while able to speed up the retraction of anterior teeth (Erdur et al., 2021; Karakasli and Erdur, 2021; Barhate et al., 2022; Ammar et al., 2024).

## 7 Conclusion

Development in age decelerates the OTM speed, and impacts the orthodontic side effects including root resorption and orthodontic pain, which is a result of altered PDL remodeling and bone turnover abilities. The cellular status, cytokine expression and tissue structural changes all contributes to the age-related changes during the orthodontic process. Current researches on age-related changes in orthodontics proves to be highly heterogeneous and are mostly focus on phenotypic changes, while the intrinsic mechanisms are not yet thoroughly understood. In order to provide guidance for accelerating tooth movement in adult orthodontics and reducing treatment side effects, more high-quality, comprehensive researches are essential.
